# Target-Based Radiosensitization Strategies: Concepts and Companion Animal Model Outlook

**DOI:** 10.3389/fonc.2021.768692

**Published:** 2021-10-20

**Authors:** Matthew R. Berry, Timothy M. Fan

**Affiliations:** ^1^ Department of Veterinary Clinical Medicine, University of Illinois at Urbana-Champaign, Champaign, IL, United States; ^2^ Cancer Center at Illinois, University of Illinois at Urbana-Champaign, Urbana, IL, United States

**Keywords:** comparative oncology, radiotherapy, dog model, molecular targets, DNA damage response

## Abstract

External beam radiotherapy is indicated in approximately 50-60% of human cancer patients. The prescribed dose of ionizing radiation that can be delivered to a tumor is determined by the sensitivity of the normal surrounding tissues. Despite dose intensification provided by highly conformal radiotherapy, durable locoregional tumor control remains a clinical barrier for recalcitrant tumor histologies, and contributes to cancer morbidity and mortality. Development of target-based radiosensitization strategies that selectively sensitizes tumor tissue to ionizing radiation is expected to improve radiotherapy efficacy. While exploration of radiosensitization strategies has vastly expanded with technological advances permitting the precise and conformal delivery of radiation, maximal clinical benefit derived from radiotherapy will require complementary discoveries that exploit molecularly-based vulnerabilities of tumor cells, as well as the assessment of investigational radiotherapy strategies in animal models that faithfully recapitulate radiobiologic responses of human cancers. To address these requirements, the purpose of this review is to underscore current and emerging concepts of molecularly targeted radiosensitizing strategies and highlight the utility of companion animal models for improving the predictive value of radiotherapy investigations.

## Introduction

Radiotherapy is instrumental in treating many cancer types and can be used in curative intent treatments alone or in combination with surgery, chemotherapy, immunotherapy, and hormone therapy. In the setting of advanced cancer, radiotherapy can be used to stabilize and provide analgesia to patients, such as in the management of skeletal metastasis. Overall, it is estimated that external beam radiotherapy is indicated in approximately 50-60% of human cancer patients (1, 2). Owing to the diverse utility of radiotherapy in curative intent protocols or palliative settings, a significant number of cancer patients can benefit from new strategies that improve radiotherapy effectiveness. Technological innovations over the last few decades have improved radiotherapy efficacy by more precise deposition of radiation energy into tumorous lesions, while decreasing exposure of the normal surrounding tissue (3). Radiotherapy has also been successfully combined with chemotherapy, termed chemoradiotherapy, and serves as first-line treatment for many human cancers including head and neck cancer, brain cancer, and lung cancer (4). Chemoradiotherapy affords spatial cooperation, with radiotherapy directed at the primary tumor site and chemotherapy targeting metastatic cancer cells. Certain chemotherapeutics can also enhance the sensitivity of cells to radiotherapy, and this radiosensitization effect has become more leverageable with the ability to provide highly conformal radiotherapy. Despite these advancements, chemotherapy-induced radiosensitization is constrained by normal tissue toxicity within the irradiated field.

With intent to improve radiotherapy responses, considerable focus has been to identify mechanistic and specific molecular targets to sensitize tumor tissue to radiotherapy. The clinical success of targeted radiotherapy is dependent on a thorough understanding of molecularly-driven radiobiologic responses, detailed preclinical investigation and appropriate clinical trial design. Despite recognizing these necessary benchmarks, many targeted radiotherapies end in disappointment, failing to provide favorable outcomes in clinical trials. In general, the failures observed in oncology clinical trials suggests the need to improve molecular target evaluation and standard preclinical modeling systems. This review will highlight several targets for radiosensitization, the necessity of appropriate *in vivo* modeling systems, and emphasize key qualities of preclinical animal models that would be expected to increase the predictive value of investigations involving radiotherapy.

## Perturbing DNA Damage Response and DNA Repair

**Figure 1:**
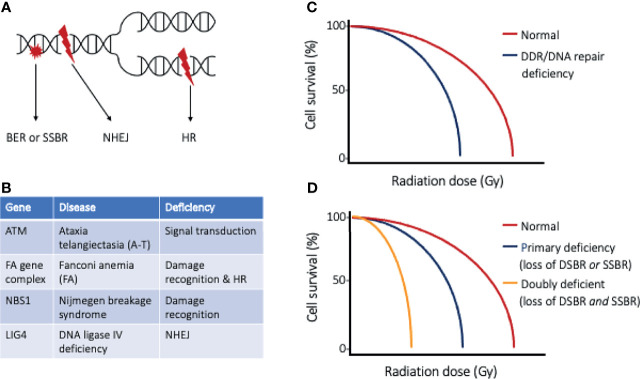
The DNA damage response and DNA repair. **(A)** Ionizing radiation results in DNA base damage, single-strand breaks, and double-strand breaks that are repaired using base excision repair (BER), single-strand break repair (SSBR), or double-strand break repair (DSBR), respectively. The types of DSBR include non-homologous end-joining (NHEJ), which can occur through all phases of the cell cycle, or homologous recombination (HR), which requires cells to have completed synthesis phase. **(B)** Table of DNA repair disorders (gene deficiencies) that are associated with clinical radiation sensitivity, and **(C)** cell survival curves of normal cells and cells with such DDR/DNA repair deficiency. Cells with defective DDR/DNA repair pathways are more sensitive to radiation injury, as exemplified by a narrow shoulder region of the survival curve and lower radiation dose to achieve lethality. **(D)** Cell survival curves highlighting the effect of targeted strategies directed at inhibiting the “back-up” repair pathway. An example of doubly deficient cells could be BRCA1/2 deficient tumor cells (loss of DSBR) and selective PARP inhibition (loss of SSBR).

Every cell within the body experiences an astonishing degree of genotoxic stress each day (5), threatening genomic integrity and risking the development of cancer. The DNA damage response (DDR) is a collection of intricate and evolutionary conserved signaling pathways that are essential to detect sites of DNA damage and facilitate DNA repair (6, 7). The DDR, when activated, halts cell cycle progression and allows for DNA to be effectively repaired, preventing the transfer of altered DNA to daughter cells. When DNA damage is severe and cannot be faithfully repaired, the DDR induces cell death through apoptosis. Ionizing radiation (IR) damages many biomolecules within cells, with DNA damage being the most notable link to radiation-induced cytotoxicity (**Figure 1A**). The DNA damage induced by IR includes base damage and single strand breaks (SSBs) that are repaired by the base excision repair (BER) and single strand break (SSB) repair pathways, respectively. The most severe forms of DNA damage induced by IR are double strand breaks (DSBs), which may be repaired through homologous recombination (HR) or non-homologous end joining (NHEJ). Cell survival following irradiation is dependent on the function of sensor, mediator, transducer, and effector molecules of the DDR, and increased sensitivity to ionizing radiation has been documented in people with germline mutations of the DDR proteins (**Figures 1B, C** **)** (8). This clinical observation has led to the development of targeted therapies that perturb the DDR, with the goal of sensitizing tumor cells to irradiation and improved radiotherapy efficacy. With defective DDR, cells progress through the cell cycle with damaged DNA, with the segregation aberrant chromosomes leading to mitotic catastrophe and ultimately cell death.

Inhibiting the function of molecules operative in the DDR, such as ataxia telangiectasia and Rad3-related (ATR), ataxia telangiectasia mutated (ATM), and DNA-dependent protein kinase (DNA-PK), have successfully sensitized cancer cells to ionizing radiation (9–12). Blocking DNA repair increases DNA damage in irradiated cells leading to increased cytotoxicity. Intuitively, the non-selective perturbation of the DDR would increase the radiosensitivity of tumor cells and normal tissues alike. Consequently, any indiscriminate increase in sensitivity of normal tissue would preclude improvement in therapeutic index and likely result in dose limitations of radiotherapy and the resultant radiotoxicity profile may limit clinical utility. Even though the DDR is not specific to tumor cells, exploitable mechanisms exist to selectivity sensitize tumor cells compared to normal surrounding cells. One way to target tumor cells is by leveraging their rapidly dividing nature and considering the mechanism of DSB repair. HR is a highly accurate DSB repair pathway, however this process requires a sister chromatid to serve as a template to repair the DNA damage. Therefore, tumor cells that are in the S or G2 phase of the cell cycle can attempt to repair radiation-induced DSBs by HR (13). Owing to the rapidly dividing nature of cancer cells, inhibition of HR is expected to differentially sensitize tumor cells more so than normal tissues that are not rapidly dividing.

More sophisticated and selective targeting of tumor cells can be achieved by performing strategic molecular profiling, highlighting specific targets of the DDR and DNA repair pathways to enhance the radiosensitivity of tumor tissues. Impaired cell cycle regulation is a common feature of cancer and most often exemplified by mutated or altered regulatory status of p53, which hampers the tumor cells’ ability to respond to DNA damage. In cells with functional p53, DNA damage results in G1/S checkpoint activation and arrest; allowing time for DNA damage to be faithfully repaired prior to DNA synthesis, and preventing accumulation of DNA damage and its mutagenic consequences. In p53 deficient tumor cells, the G1 checkpoint is not activated, and chromosome replication is initiated despite the existence of DNA damage. With the cell cycle continuing unabated, inhibition of SSB repair (such as PARP inhibition) would ultimately result in augmented DSBs in tumor cells as the replication fork meets the sites of SSBs (14). Additionally, similar to synthetic lethality in which a “doubly deficient” cell is lethal, tumor cells that have doubly perturbed SSB and DSB repair pathways are expected to suffer greater DNA damage following irradiation (**Figure 1D**). Underscoring these vulnerabilities, it has been demonstrated that cytotoxicity generated in tumor cells with SSB repair inhibition and subsequent increase in DSB formation is exaggerated in tumors already deficient in HR (such as BRCA1/2 or RAD51 mutations) (15, 16). With recognition of these co-dependent pathways, molecular profiling can allow for the rational selection of PARP inhibition strategies for tumors identified to have G1/S checkpoint disturbances and deficient DSB repair pathways. Alternatively, targeted therapies perturbing the G2/M checkpoint can be rationally selected when tumor cells are deficient in the G1/S checkpoint. Cells lacking a functional G1/S checkpoint rely more heavily on other checkpoints to avoid premature mitotic entry and segregation of mutated chromosomes. Checkpoint kinase 1 (Chk1) is an essential protein in G2 checkpoint activation (17, 18), and the disruption of the ATR-Chk1 axis can force checkpoint deficient tumor cells to proceed through the cell cycle harboring lethal DNA damage caused by IR (11, 19), with consequent activation of cell death programs. Differentially, normal tissues in the irradiated field, with normal G1/S checkpoint function, would be spared from apoptotic pathway activation.

Drawing upon analogous strategies used to identify druggable targets that mitigate the development of chemotherapy resistance, the targeted disruption of molecular radioresistance mechanisms can serve as a synergistic avenue for improving radiotherapy efficacy. Enhanced DNA repair mechanisms may allow tumor cells to resist injury by IR. Overexpression of factors involved in NHEJ (20–23) and HR (24, 25) have been documented in various cancers types, though pan-inhibition strategies to disrupt NHEJ and HR responses may hinder DSB repair in tumor cells and normal tissue leading to a narrow therapeutic window. Similarly, AP endonuclease 1 (APE1), a protein traditionally implicated in BER, has been shown to be overexpressed in tumor tissues compared to normal cells (26). The basis of developing APE1 inhibitors has been to overcome the repair capacity following genotoxic treatment. This is illustrated by the association of nuclear APE1 overexpression in head and neck cancer with poor clinical outcome and resistance to IR (27). More specific radiosensitization has been described for tumors overexpressing POLQ (also known as POL), a DNA polymerase that is error-prone and appears to provide a survival advantage for some cancers. POLQ overexpression has been shown to be a negative prognostic indicator in human cancers, including breast cancer (28, 29) and ovarian carcinomas (30). POLQ serves a role in end-joining of DSBs, separate from the canonical NHEJ described above. This alternative-end joining [also known as error-prone microhomology-mediated end-joining (MMEJ)] directed by POLQ occurs in cells when components of the canonical NHEJ are absent or fail to recognize the sites of DSBs (31). Intriguingly, POLQ has RAD51 binding motifs that hinder HR and a synthetic lethality relationship exists between HR and POLQ-mediated repair (30). Therefore, POLQ inhibition strategies for tumors with POLQ overexpression and deficiency in HR are expected to sensitize tumor cells to radiotherapy while sparing normal tissue.

## Modulating Signal Transduction Pathways

Cells possess signaling pathways that govern cell survival, growth, and proliferation. Signals may be received at the cell surface, or may arise intracellularly, and the response follows a cascade of events in the cytoplasm and nucleus of the cell. Signaling pathways are often dysregulated in cancer cells and these aberrant pathways have been investigated for targeted therapy and the development of personalized medicine. Key signaling pathways harboring potential targetable options to improve radiotherapy response include the PI3K-AKT, MAPK, and NFĸB pathways. A common theme among these pathways is their dysregulated activation favors apoptosis resistance in irradiated cells (32–34). Interestingly, several signal transduction pathway disturbances are associated with DNA repair mechanisms and will be discussed in each section.

### PI3K-AKT Pathway

The PI3K-AKT pathway is traditionally known as a cell survival pathway, though it also plays a role in cell growth and proliferation. In this pathway, Ras phosphorylates and activates PI3K leading to the formation of PIP3 on the inner leaflet of the plasma membrane. AKT recognizes PIP3 and ultimately becomes phosphorylated, activating its kinase function. This pathway can be dysregulated by multiple different mechanisms, and tumor cells with activating mutations in Ras are radioresistant as a consequence of PI3K-AKT pathway activation (35, 36). Alternatively, the loss of PTEN function (negative regulator of PI3K), PI3K activating mutations, and overexpression of receptor tyrosine kinases (such as EGFR) are other ways the PI3K-AKT pathway can be overactivated, leading to radioresistance (37). While the mechanisms by which inhibitors of the PI3K-AKT pathway promote radiosensitivity remain to be fully elucidated, existent evidence suggests that DNA repair is impaired following pathway inhibition, and renders cells more sensitive to IR (38).

Translating these foundational radiobiologic investigations to clinical practice, PI3K inhibition in a human breast cancer study resulted in HR deficiency by downregulating BRCA expression (39). This led to proposing the dual inhibition of PI3K and PARP as a synthetic lethality approach. Furthermore, tumors with loss of function mutations of PTEN have reduced HR capability, associated with reduced RAD51 expression, also suggesting the utility of PARP inhibitors (40). The dual inhibition of RAD51 and PARP sensitized tumor cells with wild type PTEN, confirming this as a targeted strategy (41). As mentioned above, cells doubly deficient in SSB and DSB repair are expected to suffer greater DNA damage following irradiation.

In addition to the activation of the PI3K-AKT by EGFR, ionizing radiation can cause EGFR, without activating its kinase domain, to be imported into the nucleus where it can directly bind and activate DNA-PK leading to DSB repair by NHEJ (42, 43). Interestingly in laboratory studies, cells pretreated with an antibody directed against EGFR (cetuximab) inhibited nuclear translocation and successfully increased their radiosensitivity (43). This unique nuclear action of EGFR suggests that tyrosine kinase inhibition (TKI) may not be sufficient to radiosensitize tumors with EGFR overexpression and other proteins required for EGFR translocation into the nucleus should be furthered explored as druggable targets (44).

### MAPK Pathway

In the MAPK pathway, ligand binding to a receptor tyrosine kinase (RTK) leads to a series of phosphorylation events from Ras → Raf → Mek → Erk1/2, which then results in the activation and production of transcription factors (inclusive of Fos and Jun) that stimulate cell growth and proliferation. There is conflicting evidence about the significance of the MAPK pathway in modulating radiotherapy response, with some evidence suggesting no role of the MAPK pathway (35), whereas other reports suggest overactivation of this pathway appears to regulate cell survival by modulating DSB repair mechanisms. Erk has been described to be a positive regulator of ATM, and thus increases DNA repair ability (45). Moreover, both NHEJ and HR repair mechanisms can be increased in tumor cells with apparent MAPK pathway activation (46, 47); and therefore inhibition of aberrant MAPK pathway activation in tumor cells may decrease their DNA repair ability and improve radiotherapy efficacy.

### NFκB Pathway

The nuclear factor-kappa B (NFκB) signaling pathway plays critical roles in inflammation, immune function, and has implications in the development and progression of cancer (34). (NFκB) is a transcription factor that is negatively regulated by (IκB). In the absence of signaling, IκB binds to and retains (NFκB) in the cytoplasm, preventing its nuclear translocation and transcriptional activity. Following stimulation, (IκB) can be phosphorylated (through the action of IKK) marking it for destruction, and (NFκB) is released and enters the nucleus to activate various target genes. (NFκB) leads to the expression of anti-apoptotic proteins including Bcl-2 and inhibitor of apoptosis proteins (IAPs) and this effect along with concurrent mitogenic signaling highlights the ability of this pathway to contribute to cancer pathogenesis. IR was previously found to increase the signaling of this pathway as a protective response, making it a prime target in radiosensitization strategies (48). Multiple investigations have shown improved radiotherapy responses following (NFκB) signaling inhibition (49–51). Additionally, the (NFκB) pathway may be activated and thus contribute to radioresistance through the action of other aberrant signaling pathways, including the PI3K/AKT. Blocking the cooperative activity of these converging pathways may provide an alternative strategy of regulating (NFκB) signaling (52).

### Other Signaling Pathways

While the mechanisms remain to be fully elucidated, other signaling pathways commonly dysregulated in cancers may be targets of radiosensitization strategies. (TGFβ) exerts pleiotropic and complex actions in cancer cells; however, there is evidence that (TGFβ) inhibition can impair DDR, possibly through reduction of ATM kinase activity (53). Pathways often associated with cancer stem cell properties, including NOTCH and Wnt/β-catinin, have also been targets of radiosensitizing strategies (54–57). It should be expected that novel mechanisms to modulate key signaling pathways will be discovered as the intersection of cancer and radiation biology continue to expand through ongoing and future research efforts. As an example, increased knowledge of the molecular pathways that are involved in the response to hypoxia have revealed the role of the unfolded protein response (UPR). Under hypoxic conditions, which is common in tumors, the UPR is activated and this in turn enhances autophagy allowing cells to better cope with hypoxic stress (58). Therefore, inhibitors of the UPR or autophagy render tumor cells more radiosensitive by decreasing their hypoxic tolerance (58, 59).

## Modifying the Tumor Microenvironment

**Figure 2:**
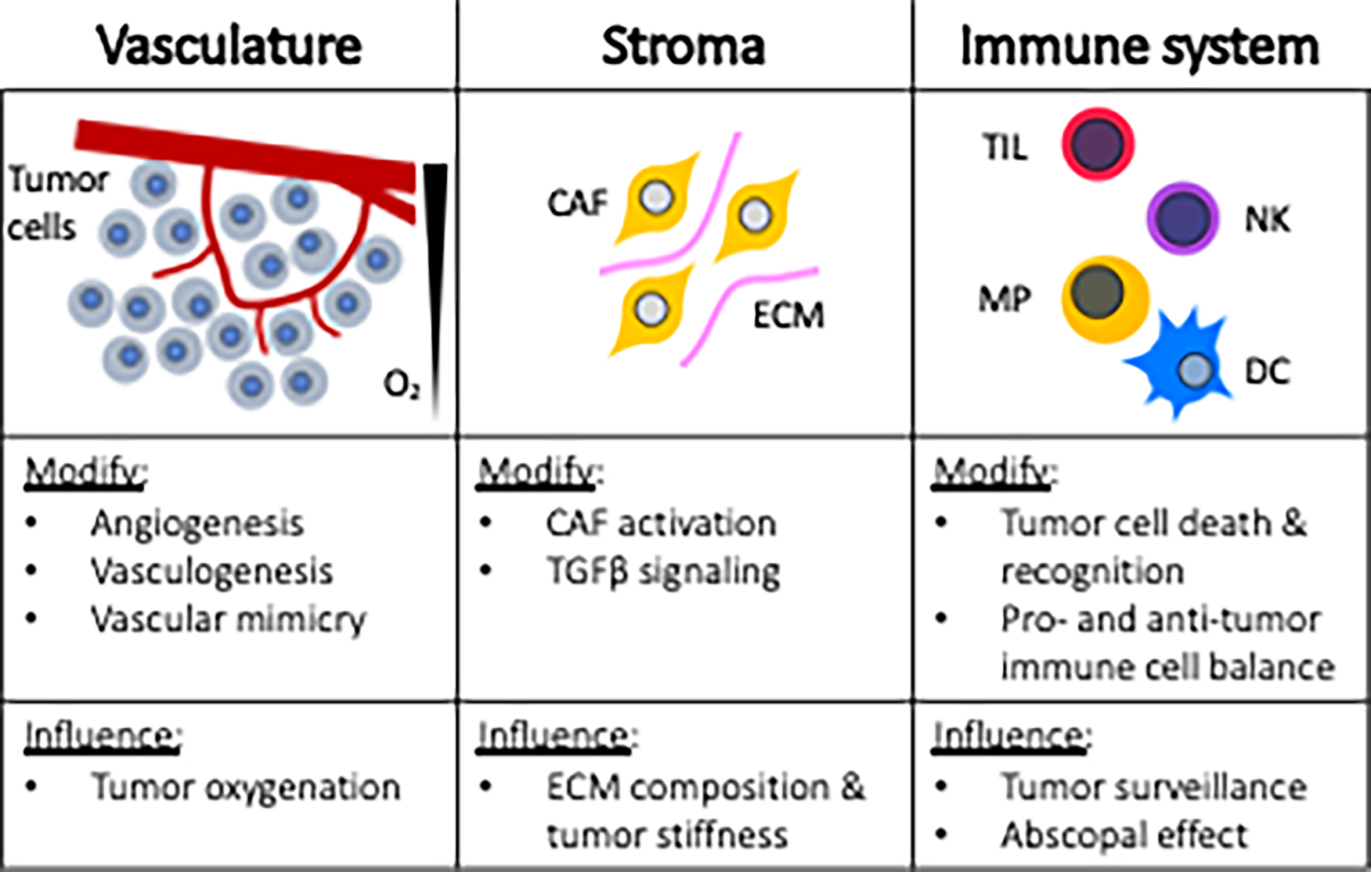
Microenvironmental targets for radiosensitization strategies. CAF, cancer associated fibroblast; ECM, extracellular matrix; TIL, tumor infiltrating lymphocytes; NK, natural killer; MP, macrophage; DC, dendritic cell.

Research targeting tumor cells directly has traditionally served as the cornerstone strategy for improving radiotherapy response. This approach may be in part attributed to the ease of performing *in vitro* experiments and highly manipulatable murine models. However, over the last few decades, a broader horizonal view for improving radiotherapeutic outcomes has been realized based upon the understanding that tumors are complex tissues containing multiple cell types that participate in heterotypic interactions. These scientific revelations now recognize the importance of the tumor microenvironment (TME) in cancer treatment strategies. **Figure 2** provides an overview of tumor microenvironmental targets, including vasculature, stroma, and the immune system. By reviewing these topics, awareness can be heightened regarding the myriad of microenvironmental targets that can be leveraged for improving anticancer radiotherapy responses.

### Vascular and Hypoxia Effects

The most extensive investigation of the TME revolves around tumor vasculature and hypoxia. Intra-tumoral hypoxia dynamically evolves during tumor growth and the distribution of hypoxia is determined by the distance to the nearest perfused capillary, the interstitium composition, and oxygen consumption by cells. These factors contribute to diffusion-limiting, or chronic hypoxia. Another form of hypoxia in tumors, referred to as perfusion-limiting or acute hypoxia, occurs as a consequence of having haphazard and abnormal structure of vasculature. Hypoxia occurs frequently in solid tumors and hypoxic cells within tumors are up to three-fold more radioresistant (60, 61). The lack of oxygen reduces the production of reactive oxygen species (ROS) following IR, ultimately decreasing radiation-induced DNA damage and consequent cancer cell death. In the absence of robust ROS generation, the induction of sublethal damage may then contribute to the tumor cell genomic instability leading to additional radiotherapy resistance. Furthermore, the degree of hypoxia has been shown to negatively influence prognosis and radiation response in multiple human cancers, including head and neck (62–64), uterine (65, 66), cervical (67), and prostate cancer (68). The association of hypoxia and prognosis serves as the foundation for investigating underlying mechanisms of hypoxia-mediated radioresistance and guides the development of targeted treatments. The strategic targeting of the hypoxic response intuitively appears to be selective to tumors, given that hypoxia is not a normal physiologic limitation in healthy non-cancerous tissues.

In response to hypoxia, cells stabilize hypoxia inducible factor (HIF)-1α through decreased activity of prolyl-4-hydroxylases (PDH), which regulates HIF-1α’s half-life. PDH functions to hydroxylate proline residues on HIF-1α in normoxic conditions, allowing it to be recognized by the tumor suppressor protein Von Hippel-Lindau (VHL). VHL ubiquitinates the hydroxylated form of HIF-1α, marking it for proteosomal degradation and preventing its action. The stabilized HIF-1α under hypoxic conditions binds HIF-1α and the heterodimer acts as a transcription factor, binding to hypoxia responsive elements (HRE) and increases the transcription of target genes (69). It is worth mentioning that HIF-1α levels may also be increased in cancer cells through overactivation of signaling pathways (oxygen independent mechanism), including PI3K/AKT and MAPK, or through loss of VHL (70, 71).

One of the many different target genes upregulated by HIF-1α is vascular endothelial growth factor (VEGF). VEGF is a ligand for a RTK (VEGFR) on the surface of cells. In the case of endothelial cells, VEGF signaling promotes survival and proliferation leading to angiogenesis. Tumors must build a vascular network to provide nutrients and oxygen to permit growth. Given the dependence of oxygen for radiation-induced ROS generation, it seems counterintuitive that inhibiting the VEGF signaling pathway would result in an improved antitumor effect when combined with radiotherapy. In fact, evidence suggests that inhibiting angiogenesis in preclinical models results in an increase in the oxygenation of tumors (72–74). Mechanistically, this paradoxical response has been linked to antiangiogenic vascular pruning effects (75), which essentially eliminate the aberrant tumoral microvasculature resulting in a net increase in tumoral oxygen concentration, with consequent increased oxygenation status improving radiotherapy responses. In addition to increasing oxygenation of the tumor and radiosensitization, this microvascular effect may also improve tumor response to systemic treatment by improving perfusion and enhancing drug delivery.

This notion has led to the concept of normalizing tumor vasculature to improve combination therapies (76), which remains an active area of study. Numerous strategies have been explored to mitigate VEGF signaling, including the development of blocking antibodies, “trapping” fusion proteins, and TKIs (77). The effects of these antiangiogenic strategies are somewhat transient given redundancy of angiogenic peptides and the clinical phenomena known as “rebound activation”. As such, hurdles to improve the clinical success of this combination strategy, including the optimal timing of antiangiogenic therapy in relation to radiation and evaluating other inhibitors playing a role in angiogenesis, such as inhibiting downstream effectors of VEGF signaling, remain to be fully evaluated (77). In response to antiangiogenic treatment, tumor cells have been shown to imitate tumor blood vessels. This process has been termed vascular mimicry and a review defines molecules and signaling pathways involved in this angiogenic-like behavior (78). The role that vascular mimicry has on radiotherapy response has not been well established; however, a glioblastoma investigation revealed that glioblastoma cells participate in tumor vasculature formation and contributes to radioresistance in a preclinical murine model (79). Last, vasculogenesis is an alternative mechanism of tumor vascular formation that appears to be driven by tumor hypoxia. Vasculogenesis involves the formation of tumor vasculature from cells primarily derived from the bone marrow (80). HIF-1α leads to the expression of CXCL12 in the TME, which acts as a chemotactic factor for bone marrow derived cells (BMDCs). Vasculogenesis has been thought to be a critical step to promote tumor growth following radiotherapy and its specific inhibition effectively prevented local tumor recurrence in a preclinical murine model (81).

Interestingly, HIF-1α activity increases following radiotherapy as a result of tumor reoxygenation and the formation of reactive oxygen species. The resulting increase in HIF-1 target gene expression has been shown to promote endothelial cell radioresistance, and HIF-1α inhibition enhanced radiotherapy response due to enhanced vascular destruction (82). This suggests HIF-1α blockade strategies can promote radiosensitization through anti-vascular effects (83). This is not the only role HIF-1α plays in regulating tumor response to radiotherapy. Deletion of HIF-1α has sensitized cancer cells to radiotherapy, and this suggests HIF-1α promotes radiation resistance in a cell autonomous manner (84). The targeting of non-malignant cells within the TME, such as the tumor vasculature and cells involved in angiogenesis or vasculogenesis, has the distinct advantage of uniform targetability compared to tumor tissues that are genomically unstable and heterogeneous in nature. Nevertheless, it is expected that further investigation of additional HIF-1 target genes [reviewed in (70)], such as those involved in cell survival, metabolism, ROS modulation, and apoptosis will identify more targeting strategies for intrinsic tumor cell radiosensitization.

### Stromal Effects

Representing an interesting and underdeveloped field is the influence of radiotherapy-induced tissue stiffness on tumor cell biology. Cells are continuously exposed to physical forces and they respond by modifying their own behavior and remodel the surrounding environment (85). Such “outside-in” signaling circuits operative in cancer cells and their immediate surroundings highlight the interfacial connectivity that exist between the extracellular matrix (ECM) and intracellular signaling responses. A review discusses ECM remodeling within tumors and the mechanisms whereby tumor stiffness can modulate tumor cell proliferation, migration, and invasion (86). Tumor stiffness, or rigidity, is determined by the composition of extracellular matrix (ECM) components. Central to the production of ECM components are cancer-associated fibroblasts (CAFs) and TGFβ signaling. Radiotherapy induces TGFβ signaling and a review discusses the action of this multifaceted cytokine in terms of radiotherapy response (87). TGFβ has been identified as a major contributor of fibrosis, or abnormal deposition of collagen and other extracellular matrix components. Therefore, instances where locoregional treatment is not curative, the alteration of ECM caused by radiotherapy may contribute to a more malignant phenotype. Proposed targets to mitigate the process of radiation-induced stiffening include inhibiting TGFβ signaling pathways and other processes leading to the activation of CAFs, including other cytokines within the TME. Limiting radiation-induced tumor stiffness may not only favorably alter tumor biology, but could also have the added benefit of enhancing drug delivery to the TME and may limit morbidity or altered quality of life that some patients experience following radiotherapy treatment.

### Immunological Effects

Modulating the immunologic responses of radiotherapy is of great interest as recent investigations have not only shown improved locoregional control, but also systemic effects that result in clinically meaningful anti-cancer immune activities. The systemic effect, or radiation-induced regression of cancerous lesions distant from the primary site of radiotherapy, is referred to as the abscopal effect, and antitumor immune cell activation is central to this phenomenon. Irradiated cancer cells release their tumor antigens that may then be taken up by antigen presenting cells (APCs) and be presented to effector T cells. In a form of cell death referred to as immunogenic cell death, irradiated tumor cells display and liberate molecules (damage associated molecular patterns, DAMPs) to enhance this APC activation. The key immune activating molecules liberated or displayed by tumor cells during radiation-induced immunogenic cell death include calreticulin, high mobility group box protein 1 (HMGB1), and ATP (88–92). The immune activating potential of radiotherapy heightens interest in developing and optimizing concurrent strategies of localized immune activation within the TME as an *in situ* vaccine strategy (93).

Perhaps the best studied and most exciting combinations involving immunotherapy and radiotherapy are checkpoint blockade strategies. Checkpoint inhibitors currently in use are immunomodulatory antibodies, and are so named because they block normal negative regulators of T cell immunity, consequently removing their brake system (94). The targets of current checkpoint inhibitors include CTLA4 and PD1/PD-L1. This immunotherapy strategy exerts monotherapy activity in different types of malignancies; however, not all patients experience significant clinical benefit. This observation has paved interest for future studies to investigate predictive biomarkers and to evaluate checkpoint blockade with other treatment modalities (95). Particularly interesting is the combination treatment with radiotherapy where remarkable abscopal effects have been observed (96–98).

Evasion of immune recognition is a hallmark of cancer (99), and this can be accomplished in multiple ways. Downregulation of MHC class 1 molecules on cancer cells is one such strategy, making tumor cells undetectable by CD8^+^ cytotoxic T lymphocytes (CTLs) (100). Studies have shown that radiation therapy can increase MHC class 1 expression to help overcome this immune-evasion strategy (101–103). While it is clear that radiotherapy can have immunostimulatory roles, there are also immunosuppressive actions that must be recognized and overcome. HIF stabilization, secondary to inherent tumor hypoxia or as a response to radiotherapy, can promote immune evasion through the expression of CD39, CD47, CD73, and PDL1 (104). Thus, perturbing HIF activity may reduce the immunosuppressive consequence of radiotherapy. It has also been recognized that HIF-1α can actually improve function of some immune cells; therefore, exploration of HIF modulation in terms of improving immune recognition of cancer is an active area of research (105).

The contrasting immunostimulatory and immunosuppressive effects of radiotherapy are related to the co-existence of pro-tumor and anti-tumor immune cells within the TME. CTLs, M1 macrophages, and natural killer (NK) cells are considered to be the most effective anti-tumor cells within the TME; whereas pro-tumor immune cells include T regulatory cells (Tregs) and M2 macrophages (106). Multiple factors can contribute to a shift in the balance of these immune cells in response to radiotherapy, which may include their inherent radiosensitivities or alteration of cytokine profiles within the TME. Investigations have shown a relative increase in the immunosuppressive immune cell profile following radiotherapy (107–110). A notable cytokine is TGFβ, which is known to be upregulated by radiotherapy and has pronounced immunosuppressive effects (87). Briefly, this cytokine can be secreted from CAFs, dying cancer cells, or specific immune cells and it has been found to influence many of the cells within the TME. For immune cells, TGFβ tends to favor formation of Treg cells and the transition of macrophages into the M2 phenotype. Additionally, TGFβ appears to inhibit the release of IL-2 which subsequently prevents proliferation of CTLs and NK cells. Therefore, the effects of TGFβ signaling is considered a major obstacle to overcome and numerous TGFβ blockade investigations are underway to leverage the beneficial immunological effects of radiotherapy (111).

## Utility of Companion Animal Models in Radiotherapy-Drug Development

Comparative oncology involves the study of naturally occurring cancers in nonhuman species that function as a complementary model for advancing human cancer research (112–114). Companion animal models, mainly pet dogs, are the focus of the NCI Comparative Oncology Program (https://ccr.cancer.gov/comparative-oncology-program) and there is an increasing awareness of the value of spontaneous tumor models in drug development (115). The Comparative Oncology Trials Consortium (COTC) network (https://ccr.cancer.gov/comparative-oncology-program/consortium) is made up of 22 comparative oncology centers, many of which are equipped with state-of-the-art radiotherapy technology. With such a collaborative network in place, companion animal models are uniquely positioned to advance drug-radiotherapy combination development. While strong translational value can be gained through purposeful inclusion of companion animal models in radiotherapy-drug development, studies in companion pets should not be viewed as a substitute for traditional preclinical investigations. Instead, companion animal models are expected to improve the predictive value of preclinical investigations.

**Figure 3:**
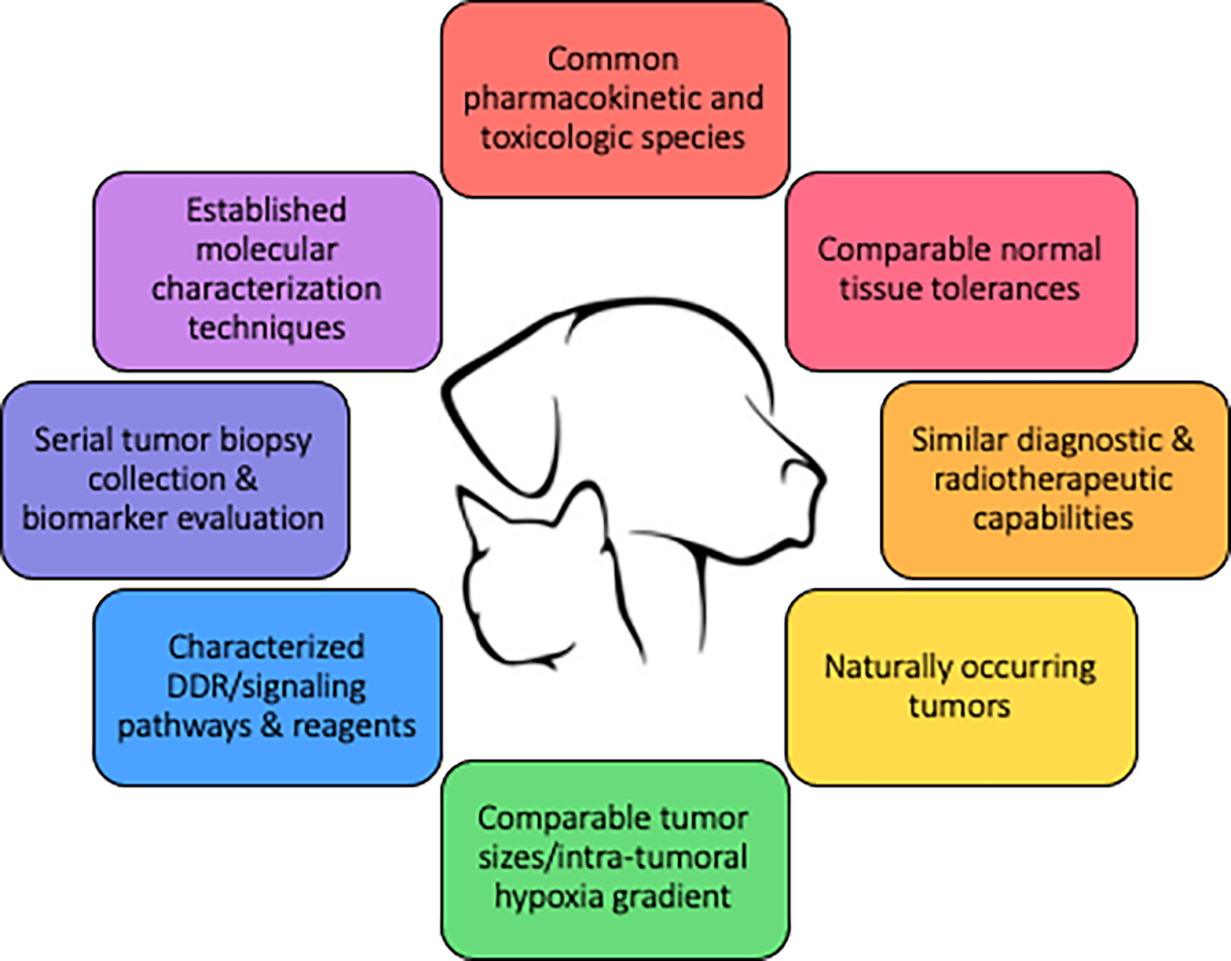
Qualities of a large animal tumor model to improve the predictive value of drug-radiotherapy combination studies.

**Figure 3** defines key qualities of a preclinical modeling system to improve the predictive value of combination therapies involving radiotherapy, and underscores how companion animals are uniquely capable of fulfilling these qualities. The spontaneous nature and the coevolution of tumor and microenvironment, tumor heterogeneity, and intact immune systems are intuitive reasons why companion animal models could better predict clinical success compared with traditional murine models. Similar radiobiologic principles and normal tissue tolerances also favor companion animals as a better predictive model, as this would allow for similar fractionation and total radiotherapy dose prescriptions. Perturbations of the DNA damage response (DDR), discussed previously, is one major focus in targeted radiosensitization strategies and dogs have already been suggested to be models of investigating DDR pathways (116, 117). The diagnostic imaging and radiotherapy capabilities available to companion animals are identical to those used in human cancer patients, allowing direct translation of methods and radiotherapy protocols into clinical trial design. Intra-tumoral hypoxia gradients, which largely influence radioresistance observed in human cancers cannot be faithfully recapitulated in preclinical murine models due to the relative size limitation in rodent species. Larger animal models, including cats and dogs, better recapitulate the intra-tumoral hypoxia gradients that naturally develop during progressive macroscopic growth as observed in human cancers.

Additional key qualities provided by companion animal models revolve around the relative ease of sample collection. Companion animals, especially the dog, are frequently used in pharmacokinetic and toxicologic studies during drug development. This information can be used to streamline the use of the tested species in efficacy and proof-of-concept investigations. For the rational selection of targeted molecular therapies, rapid data collection and interpretation of individual tumors is imperative. The COTC previously established molecular characterization techniques for dog tumors (118). The study validated workflow for prospective genetic profiling of individual canine tumors within a clinically relevant timeframe (<1 week), and this can support the use of a heterogenous population of dogs for preclinical modeling of personalized medicine. Biomarkers are needed to select patients most likely to benefit from any form of targeted therapy and to monitor response to treatment. Serial tumor biopsies can be incorporated into companion animal trials, and this can assist in biomarker identification that subsequently can be incorporated into human clinical trials.

**Figure 4:**
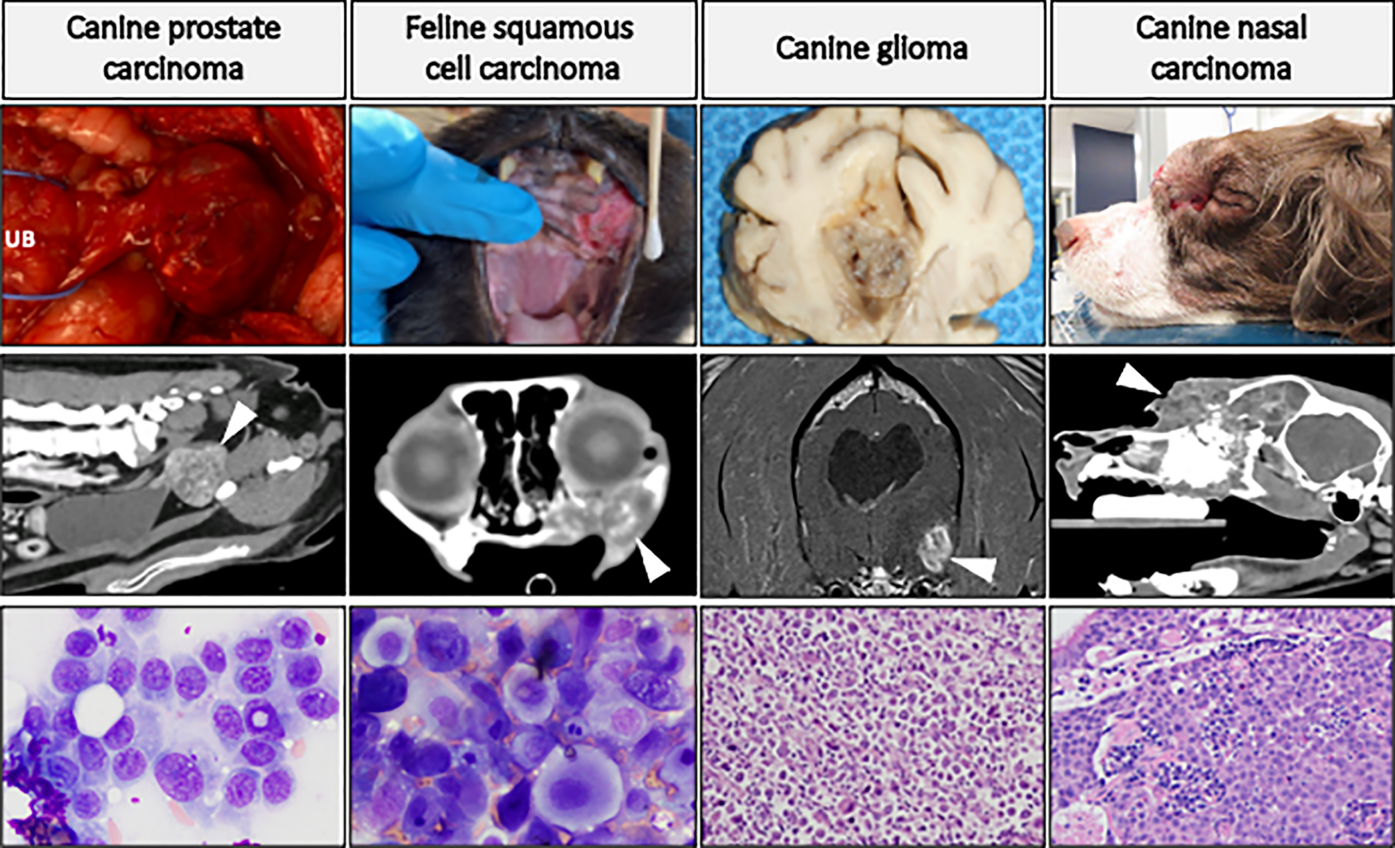
Panel of companion animal tumors that are often treated with radiotherapy. Top row: gross tumor images of canine prostate carcinoma (UB, urinary bladder), glioma, and nasal carcinoma (causing facial deformity), and feline oral squamous cell carcinoma. Middle row: advanced diagnostic imaging of respective cancer types (CT images, except for glioma which is MRI) with white arrows directed at tumor tissue. Bottom row: cytologic preparations (Wright-Giemsa) of canine prostate carcinoma and feline oral squamous cell carcinoma; histologic (H&E) images of canine glioma (high-grade astrocytoma) and canine nasal carcinoma. This is not an exclusive list of cancers with translational potential for radiotherapy investigations.

**Figure 4** highlights four companion animal cancers that are most often treated with radiation therapy, as surgical treatment is often not possible or not elected by the pet owner. The cancers displayed in this figure are not exclusive examples of cancers with translational potential in radiotherapy investigations. Instead, the panel of images are meant to provide examples of how tumors appear in companion animals, to help the reader envision how the radiosensitization strategies reviewed in preceding sections can be evaluated in companion animal cancers. Companion animals have already been used to advance radiotherapy-based investigations; with canine cancers having been defined as a translational model of tumor hypoxia (119), and dogs and cats have helped advance the understanding of how hyperthermia can be combined with radiotherapy to improve tumor control (120, 121). Radio-immunotherapy strategies have also been investigated in dogs, including checkpoint inhibition (122), modulation of the immunosuppressive microenvironment (123), and enhancing NK cell cytotoxicity (124). Readers interested in radio-immunotherapy strategies are also directed to a review exploring the potential utility of two immunogenic canine cancers (oral malignant melanoma and appendicular osteosarcoma) for maximizing immunogenic cell death and abscopal effects (125). Other radiotherapy-drug combination studies in companion animals have evaluated synergistic activity of a novel druggable target (126) and the use of radiotherapy for induction of druggable target-protein expression (127). Collectively, these examples highlight recent successes and provide the rationale for purposeful inclusion of companion animal models for improving the predictive value of radiotherapy investigations.

## Conclusions and Future Directions

This review highlights several potential molecular targets and pathways for the rational development of radiosensitizing strategies. Importantly, given the conserved DNA damage responses and tumor histologies shared between human beings and companion animals, unique opportunity is afforded by the purposeful inclusion of pet dogs and cats with naturally occurring cancers for validating investigational radiotherapeutic interventions. In addition to shared tumor histologies, the faithful recapitulation and evolution of key tumor microenvironmental factors inclusive of vasculature, immunocytes, and stroma, can be powerfully leveraged for more accurate preclinical modeling of novel radiotherapeutic protocols. Companion animals have already been used to advance the understanding of tumor radiobiology and treatment. With this proven and existing foundation, companion animals with naturally occurring cancers will likely improve the predictive value of target based radiosensitization strategies and accelerate the translation of innovative radiotherapeutics regimens in human cancer patients.

## Author Contributions

MB and TF envisioned contents of review article. MB wrote original draft and developed all figures. MB and TF revised manuscript and figures for final submission. All authors contributed to the article and approved the submitted version.

## Funding

This review article was supported by Morris Animal Foundation award D19CA-064.

## Conflict of Interest

The authors declare that the research was conducted in the absence of any commercial or financial relationships that could be construed as a potential conflict of interest.

## Publisher’s Note

All claims expressed in this article are solely those of the authors and do not necessarily represent those of their affiliated organizations, or those of the publisher, the editors and the reviewers. Any product that may be evaluated in this article, or claim that may be made by its manufacturer, is not guaranteed or endorsed by the publisher.
